# A consistent and general modified Venn diagram approach that provides insights into regression analysis

**DOI:** 10.1371/journal.pone.0196740

**Published:** 2018-05-17

**Authors:** Robert M. O’Brien

**Affiliations:** Department of Sociology, University of Oregon, Eugene, Oregon, United States of America; Jilin University, CHINA

## Abstract

Venn diagrams are used to provide an intuitive understanding of multiple regression analysis and these diagrams work well with two variables. The area of overlap of the two variables has a one-to-one relationship to the squared correlation between them. This approach breaks down, however, with three-variables. Making the overlap between the pairs of variables consistent with their squared bivariate correlations often results in the overlap of two of these variables with the third variable that is not the same as the variance of the third variable accounted for by the other two variables. I introduce a modified Venn diagram approach that examines the relationships in multiple regression by using only two circles at a time, provides a new and consistent reason why the circles need to be of the same size, and designates a “target variable” whose overlap with the other circle corresponds to the variance accounted for by the other variable or variables. This approach allows the visualization of the components involved in multiple regression coefficients, their standard errors, and the *F*-test and *t*-test associated with these coefficients as well as other statistics commonly reported in the output of multiple regression programs.

## Introduction

The use of Venn diagrams in statistics can provide a way to make concepts such as variance accounted for in the dependent variable, multiple regression coefficients, the effects of multicollinearity between the independent variables on standard errors, and associated significance tests more intuitive to students and professionals [1–6]. The traditional Venn diagram approach does this by making an analogy between the proportion of area of overlap between circles that represent two variables and the proportion of variance accounted for. This works clearly and simply for bivariate regression, but it only works sometimes in situations with two or more independent variables. This inconsistency creates problems for using the traditional Venn diagram approach to represent multiple regression problems where there are two or more independent variables.

This inconsistency limits the usefulness of the traditional Venn diagram approach. In this paper a modified Venn diagram approach is outlined that addresses the problems with the traditional approach when there are two or more independent variables. I carefully lay out the modified Venn diagram approach and show how these diagrams relate to the most commonly reported statistics associated with multiple regression. I show how the diagrams relate to the formulas for these statistics.

The modified Venn diagram approach that I propose allows for an improved understanding of regression analysis. This means giving up, however, the representation of the individual effects of the *k* independent variables in a multiple regression analysis. Dispensing with diagrams having three or more circles allows a consistent representation of the standard features of regression analysis diagrammatically. I concentrate on the standard output from multiple regression analysis programs, including; $R_{y}^{2}$, the *F*-test for the significance of $R_{y}^{2}$, the regression coefficients and their *t*-test for statistical significance, the standard errors of the regression coefficients, variance inflations factors, and the Analysis of Variance of regression table. More can be done with the proposed system, but I leave that for others to explore. Although we give up something in the process of not using a single Venn circle for each of the independent variables; we gain much by doing so. It allows us to leave behind the inconsistent representations inherent in the traditional use of Venn diagrams in statistics.

### Two examples from the traditional approach

The traditional Venn diagram approach works in the bivariate case: one independent variable and one dependent variable. For convenience of representation, each variable has the same variance and same area. This is accomplished by using standardized variables, so that each variable has a variance of one.

**Fig 1:**
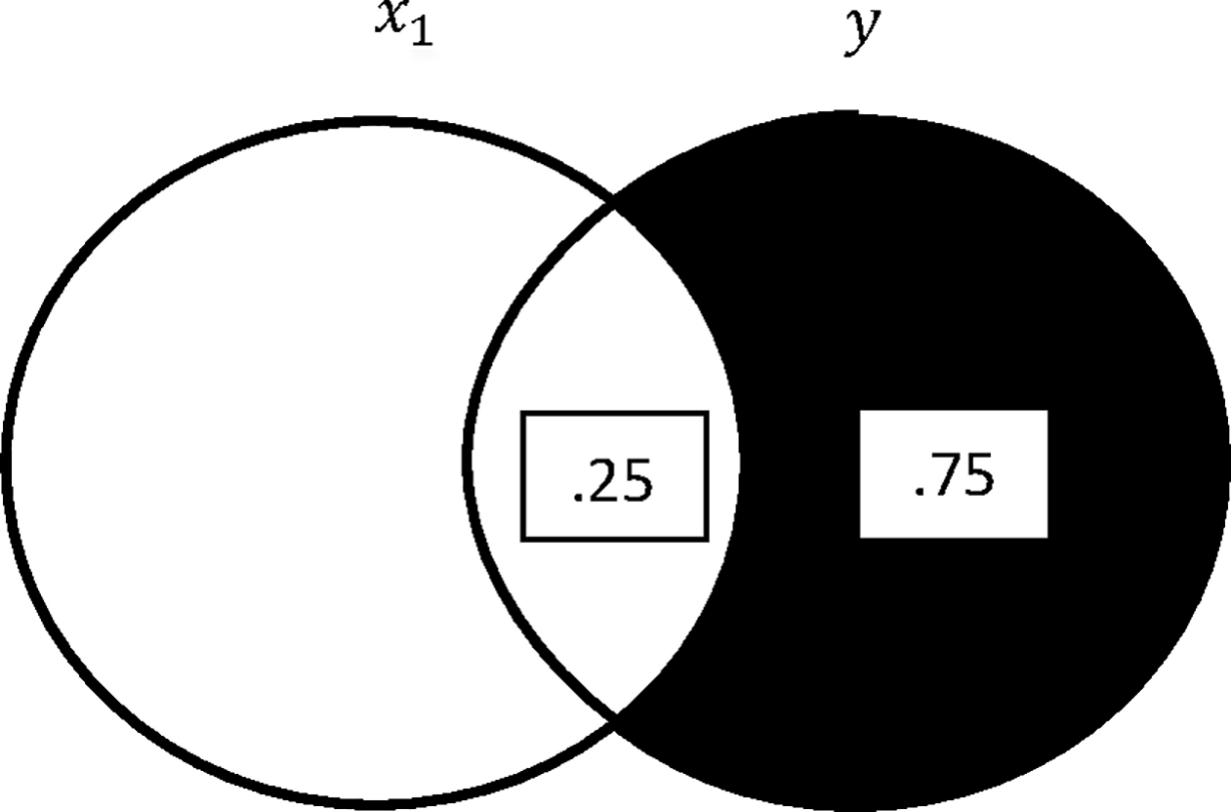
Traditional Venn diagram depicting two correlated variables (*r*_1*y*_ = .50 or −.50).

Fig 1 shows the simple bivariate situation of one independent variable (*x*) and a dependent variable (*y*). The correlation between the two variables is .50 or −.50, one cannot determine which from the diagram (the diagram works in both situations). The squared correlation coefficient is represented by the area of overlap between the two variables, which is .25 $\left(=r_{xy}^{2}=.5^{2}={-.5}^{2}\right)$. The variance that is not accounted for by *x* in the dependent variable is .75 $\left(=1-r_{xy}^{2}\right)$. The squared standardize regression coefficient of *y* regressed on *x* is the area in *y* uniquely accounted for by *x* divided by the variance in *x* that is not associated with the other independent variables in the model. Since *x* is the only independent variable in the model, the area in *y* that is associated uniquely with *x* is .25. There is no other independent variable in the model; thus, the area of *x* not associated with the other independent variables is 1.00. Therefore, the standardized regression coefficient squared is .25 $\left(\beta_{yx}^{2}=.25/1.00\right)$ and |*β*_*yx*_| = |.5|. To obtain the absolute value of the unstandardized regression coefficient, |*b*_*yx*_|, we multiply the absolute value of the standardized coefficient by (*sd*_*y*_/*sd*_*x*_), where *sd*_*y*_ is the standard deviation of *y* and *sd*_*x*_ is the standard deviation of *x*. I could go on (as we will see in the next sections), but these are the basic statistics typically discussed in the bivariate regression representations that use Venn diagrams.

This approach works well in the two-variable case (two Venn circles). Note that the diagram represents squared quantities such as squared correlations, the proportion of the variance (a squared quantity) that is accounted for by the independent variable, the proportion of the variance that is not accounted for by the independent variable, and the squared standardized regression coefficient.

**Fig 2:**
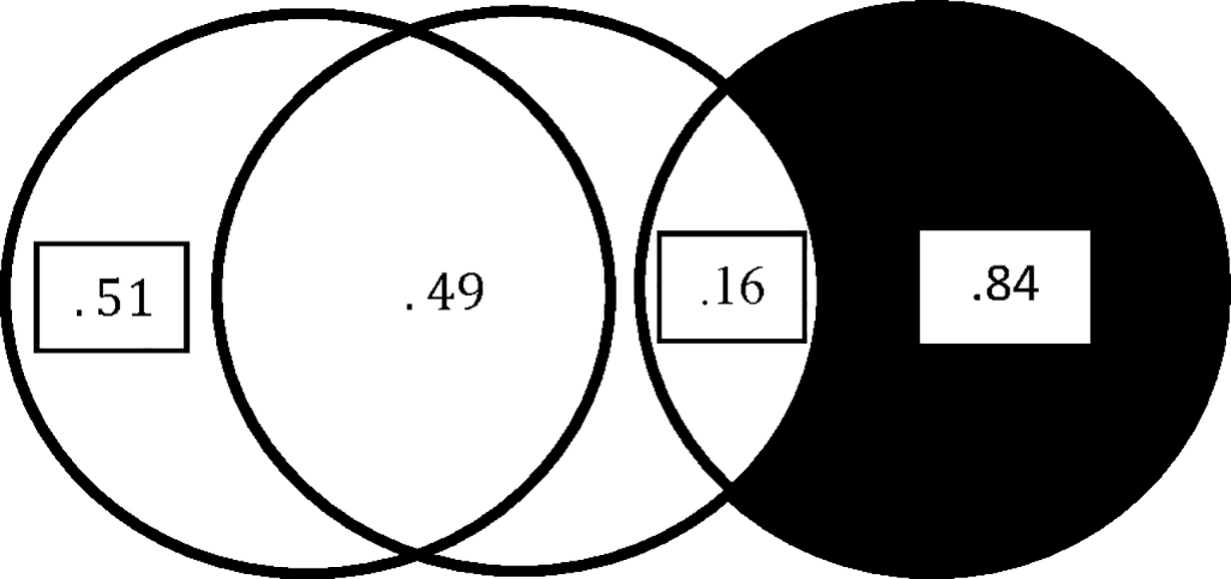
Traditional Venn diagram for two independent variables and one dependent variable: *r*_12_ = .70 or −.70, *r*_2*y*_ = 0, and *r*_1*y*_ = .40 or −.40.

Fig 2 depicts a situation in which *r*_12_ = ±.70, *r*_*y*1_ = ±.40, and *r*_*y*2_ = .00. There is no problem in presenting the *bivariate overlaps* with the Venn diagram in Fig 2. There is a fundamental problem, however, with the diagram in Fig 2 that has to do with the overlap of *x*_1_ and *x*_2_ with *y*. The total overlap of the two independent variables with the dependent variable in that diagram is .160 while $R_{y∙12}^{2}$ = .314. The combined variance in *y* accounted for by *x*_1_ and *x*_2_ is .314 not .160. This is easily verified using the equation for $R_{y∙12}^{2}$ found in many intermediate statistics texts:

$$R_{y∙12}^{2}=\frac{r_{y1}^{2}+r_{y2}^{2}-2\times r_{12}\times r_{y1}\times r_{y2}}{1-r_{12}^{2}}.$$ (1)

In our case, because *r*_*y*2_ = 0, $R_{y∙12}^{2}=\left(.16+0-0\right)/(1-.49)=.314$. The variance not accounted for by the two independent variables is .686 and not .840 as implied by the tradition Venn diagram approach. This problem with the variance accounted for in *y* by the two independent variables is a case of “suppression” [7–9], which is not handled well by Venn diagrams. In the two independent variable case, suppression occurs when the variance accounted for in the dependent variable increases when one of the two independent variables is controlled for by the other. In the present case, $R_{y∙12}^{2}>r_{y1}^{2}+r_{y2}^{2}$.

The variance shared by the two independent variables is .49 as implied by the diagram, but this shared variance for the independent variables can be misleading in the case of three independent variables. With three independent variables; for example, suppression can occur among these variables and make the total overlap of the independent variables based on their bivariate relationships to each other misleading.

### Two independent and one dependent variable using a modified Venn diagram approach

**Fig 3:**
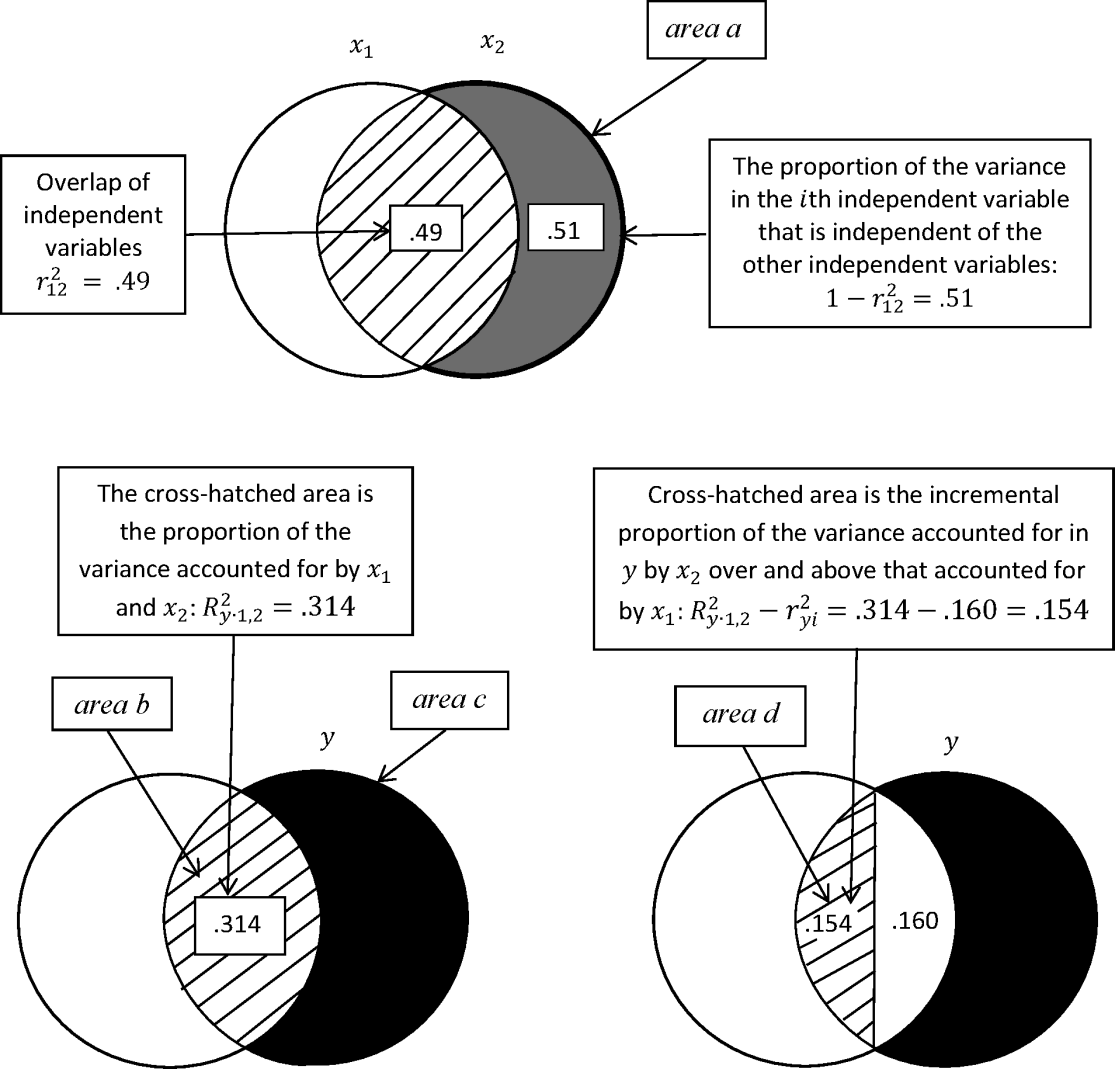
Modified Venn diagram for two independent variables and one dependent variable: *r*_12_ = ±.70, *r*_2*y*_ = 0, and *r*_1*y*_ = ±.40.

In Fig 3, I present a modified Venn diagram with the same data used in Fig 2, but this diagram is consistent in terms of overlaps and the variance accounted for in the “target variables.” I first show how this modified Venn diagram approach can be used to illustrate the regression components of most interest in the two independent variable case. In the following section, I generalize this approach to *k* independent variables. Achieving consistency between the proportions of variance accounted for and proportion of area overlapped requires modifications in the traditional approach to using Venn diagrams for statistical interpretations. These modifications include: (1) The number of circles in the diagram considered at any one time is no greater than two (see [2]) for an excellent example of what can be accomplished by concentrating on just two circles in a Venn diagram]. (2) Each of the circles has the same size, but this is not because they are standardized to have variances of 1.00. They are of the same size to represent that each variable or combinations of variables can account for a proportion of variance in the “target” variable of from 0 to 1.00. (3) In my two circle diagrams, one of the circles represents one or more variables and the other circle is considered the target variable: the target variable is the one for which the proportion of the area overlapped by the other circle is proportional to the variance accounted for in that variable. (4) We assign the overlap of the dependent variable with the combined independent variables into a portion that is uniquely associated with the independent variable of interest (the target independent variable) and a portion that is not uniquely associated with the target variable.

Circles of the same size allow for the proportion of the area overlapped in the target variable to correspond to the proportion of the variance accounted for. This innovation in the rationale for using circles of the same size is necessary because the circle representing the combined effects of *x*_1_ and *x*_2_, for example, is not a standardized variable with a variance of one. It is of the same size as the target variable so that it can overlap in area with the target variable from 0 to 1.00, which corresponds to the variance in the target variable that can be statistically associated with one or more variables. The modified Venn diagram approach provides a diagrammatic visualization of the statistical components that provide all of the standard output for basic multiple regression analyses. I focus on the basic regression analysis components to illustrate the modified Venn diagram approach.

Fig 3 contains two panels or rows of diagrams. The top panel displays the overlap of the two independent variables, the target variable (the one for which we compute the regression coefficient and associated statistics: *x*_2_). This diagram is easily handled by the traditional Venn diagram approach or this modified approach. The overlap of the target variable with *x*_1_ is .49 in the diagram and .51 of the target variables’ variance is independent of the other independent variable or not accounted for by the other independent variable in the model. We have labelled this independent or unaccounted for variance in the target independent variable as “*area a*” in the diagram.

The second panel of Fig 3, on the left-hand side, displays the *combined* overlap of *x*_1_ and *x*_2_ with *y*: the cross hatched area is the proportion of the variance in *y* accounted for by *x*_1_ and *x*_2_. This cross hatched area is .314 of the area of *y* (here *y* is the target variable and in this case the dependent variable); this area is labeled “*area b*.” This diagram represents the area of overlap, corresponding to $R_{y∙12}^{2}$, for the two independent variables correctly (which was not the case in Fig 2). The black section of *y* is the proportion of the variance of *y* that is not accounted for by the two independent variables in the model and is labelled “*area c*.” The strategy here is to show the total overlap using a single circle to represent the effects of both *x*_1_ and *x*_2_.

The diagram, to the right in this panel shows the amount of the variance in *y* explained by *x*_1_ and *x*_2_ again, but this time it is broken into a part that is associated with *x*_2_ (the cross-hatched area: “*area d*”) after allowing the other independent variable to account for all of the variance in *y* that it can account for: that is, the increment in the proportion of variance accounted for in *y* due to the addition of *x*_2_ to the model: $R_{y∙12}^{2}-r_{y1}^{2}=.314-.160=.154$. This representation is another modification in the traditional Venn diagram approach.

I now show how the modified Venn diagram approach allows us to visualize the basic statistics that are typically reported in the output of multiple regression programs. Two measure of multicollinearity for the *i*th independent variable are the tolerance and the variance inflation factor: the tolerance equals $\left(1-r_{12}^{2}\right)$ or (*area a*) in the diagram in the first panel and the reciprocal of the tolerance “the variance inflation factor” equals $1/(1-r_{12}^{2})$ or 1/*area a*. The variance in *y* accounted for by *x*_1_ and *x*_2_ is represented in the left-hand diagram in the second panel as *area b* $\left(area\;b=R_{y∙12}^{2}=.314\right)$; the cross-hatched area in the left-hand diagram. The black area in that diagram is the variance in *y* not accounted for by the two independent variables and is labelled as *area c* $\left(area\;c=1-R_{y∙12}^{2}=.686\right)$. These statistics are often reported in the output for multiple regression and correspond directly with the areas in the diagrams. Not surprisingly the diagrams that involve areas of overlap relate to squared terms in multiple regression.

$\beta_{y2∙1}^{2}$, is the squared standardized regression coefficient for *y* regressed on *x*_2_ controlling for *x*_1_ and is equal (*area d*/*area a*); that is, the increment in the proportion of the variance in the dependent variable accounted for when *x*_2_ is added to a model that contains the other independent variable divided by the proportion of the variance in *x*_2_ that is independent of the other independent variable. It is the rate of change in the proportion of the variance in *y* accounted for uniquely by *x*_2_ for a change that is equal to the proportion of the variance in *x*_2_ that is independent of the other independent variable:

$$\beta_{y2∙1}^{2}=\frac{R_{y∙1,2}^{2}-r_{y1}^{2}}{1-r_{12}^{2}}=\frac{area\;d}{area\;a}.$$ (2)

For our data, taking the square root of $\beta_{y2∙1}^{2}$ yields the absolute value of the standardized regression coefficient: $|\beta_{y2∙1}|=\sqrt{.154/.510}=.550$. Eq (2) can be derived from Eq (3.5.7) in [1].

The *F*-test for this coefficient (with the null hypothesis that the standardized coefficient is zero) is the proportion of the variance in *y* that is uniquely accounted for by *x*_2_ divided by the proportion of the variance in *y* variable that is not accounted for by the independent variables that has itself been divided by its associated degrees of freedom. This provides a significance test for $\beta_{y2∙1}^{2}$, or for *β*_*y*2∙1_, or (as we will see) the unstandardized regression coefficient *b*_*y*2∙1_:

$$F(1,n-k-1)=\frac{R_{y∙1,2}^{2}-r_{y1}^{2}}{\left(1-R_{y∙1,2}^{2})/(n-k-1\right)}=\frac{area\;d}{area\;c/(n-k-1)},$$ (3)

Where *F* has one degree of freedom associated with the numerator and (*n* − *k* − 1) degrees of freedom associated with the denominator.

The standard error for the standardized regression coefficient (*β*_*y*2∙1_) is the square root of the proportion of the variance in *y* not accounted for by the independent variables in the model that has been divided by its associated degrees of freedom divided by the proportion of the variance in *x*_2_ that is independent of the other independent variable; that is, the square root of *area c* divided by its associated degrees of freedom divided by the square root of *area a*:

$$SE(\beta_{y2∙1})=\sqrt{\frac{\left(1-R_{y∙1,2}^{2})/(n-k-1\right)}{1-r_{12}^{2}}}=\sqrt{\frac{area\;c/(n-k-1)}{area\;a}}.$$ (4)

These components for these inferential statistics are distinctly visualized in the modified Venn diagrams and offer clear intuitions into how these inferential statistics work. For example, for the *F*-test *area d* is the unique proportion of the variance in *y* associated with *x*_2_ (the unique explanatory power of *x*_2_) and *area c* is the proportion of the variance in *y* unaccounted for by independent variables $\left(1-R_{y∙1,2}^{2}\right)$: the residual or “error” proportion of the variance of *y*. The larger the unique proportion of variance accounted for by *x*_2_ and the smaller the proportion of the variance unaccounted for by the independent variables, the greater the calculated value of *F* (all else remaining the same). Importantly, *area c* in the denominator is divided by its degrees of freedom (to become the residual variance) showing the effects of the sample size and the number of independent variables on the calculated value of *F* in the Eq (4). One can easily visualize the effects of making *area c* smaller and *area d* larger (and other combinations) on the results of an *F*-test.

The most common test of the statistical significance of the regression coefficients is to use a *t*-test that calculates the value of *t* as the regression coefficient divided by its standard error:

$$t_{\left(n-k-1\right)}=\frac{\sqrt{area\;d/area\;a}}{\sqrt{[area\;c/(n-k-1)]/area\;a}}=\sqrt{\frac{area\;d}{area\;c/(n-k-1)}}=\sqrt{F_{\left(1,n-k-1\right)}}.$$ (5)

When there is one degree of freedom associated with the numerator *t* equals $\sqrt{F}$. One will get the same result using *t* or *F* to test for the statistical significance of $\beta_{y2∙1}^{2}$, or *β*_*y*2∙1_, or the unstandardized regression coefficient *b*_*y*2∙1_. Interestingly, *area a*, which represents the tolerance, drops out of the significance test. It represents multicollinearity, and its absence shows that the value of this measure of multicollinearity and its reciprocal (VIF) do not affect the significance test for the regression coefficients.

### Diagrams when there are *k* independent variables

**Fig 4:**
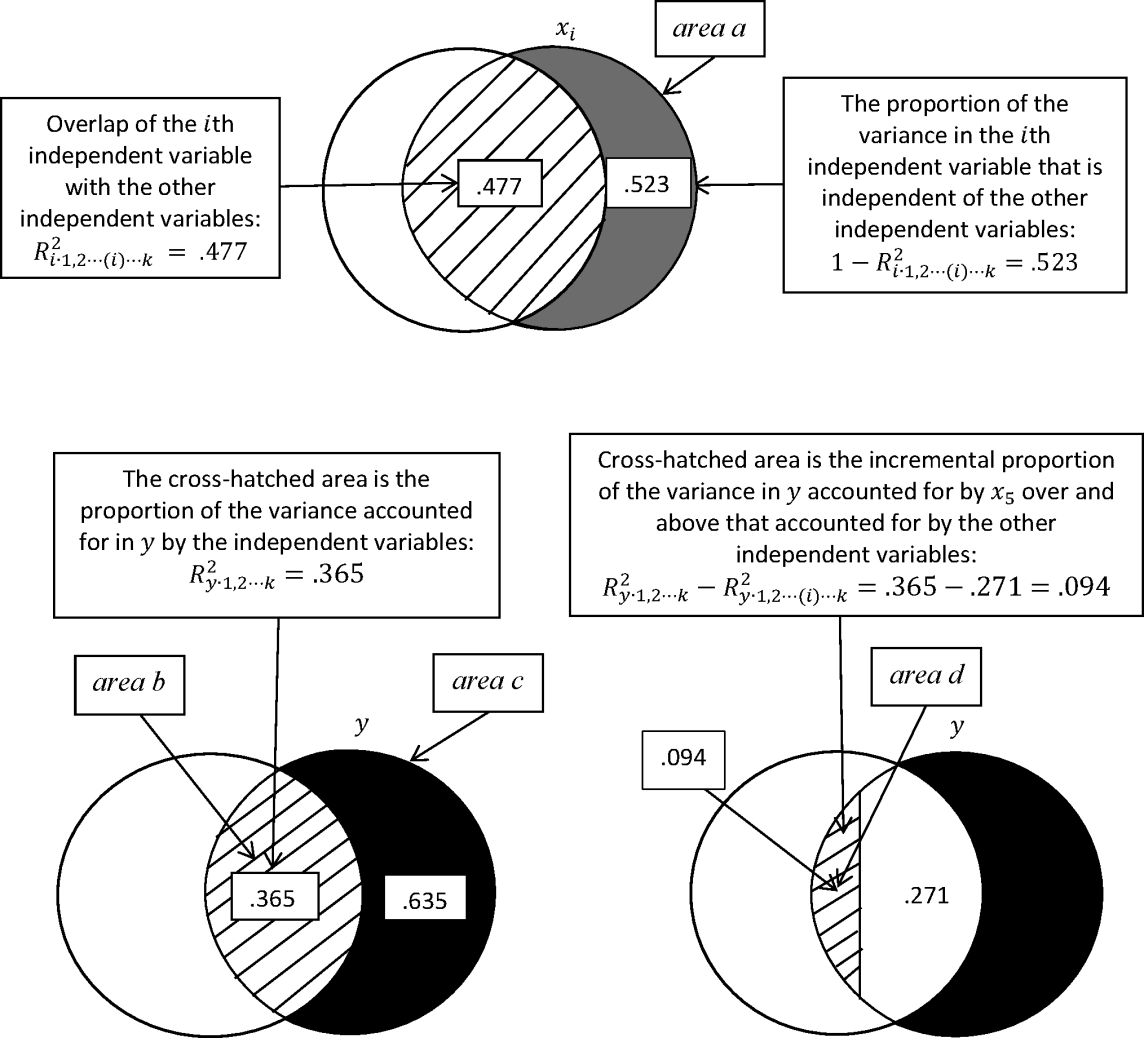
Modified Venn diagram for *k* independent variables and one dependent variable: Based on data in Table 1.

The extension to the *k* independent variable situation is straightforward for the modified Venn diagram approach. First, however, a quick note on notation is in order: $R_{y∙1,2\cdots k}^{2}$ is the multiple correlation coefficient squared for *y* regressed on all *k* of the independent variables. $R_{y∙1,2\cdots (i)\cdots k}^{2}$ is the multiple correlation coefficient squared for *y* regressed on all *k* of the independent variables except for the *i*th independent variable, which can be any one of the *k* independent variables. $R_{i∙1,2\cdots (i)\cdots k}^{2}$ is the multiple correlation coefficient squared between the *i*th independent variable and the other independent variables in model. The (*i*) notation, which indicates the absence of the *i*th variable in the list, is also used with standardized regression coefficients $\beta_{yi∙1,2\cdots (i)\cdots k}^{2}$. In Fig 4, we treat *x*_5_ (the fifth independent variable) as the *i*th independent variable, so that *i* will represent this fifth independent variable (the independent variable of interest) in that figure.

Table 1 contains the correlation matrix that we work with to construct the overlapping areas in the diagrams in Fig 4. In this example, I calculate the basic statistics that were presented in the previous section, and I show how the analysis of variance of regression corresponds to the modified Venn diagrams.

**Table 1:** Correlation matrix for five independent variables and one dependent variable.

	*x*_1_	*x*_2_	*x*_3_	*x*_4_	*x*_5_	*y*
*x*_1_	1	0.6	0.5	0.2	0.2	0.2
*x*_2_	0.6	1	0.7	0.2	0.3	0.25
*x*_3_	0.5	0.7	1	0.2	0.2	0.4
*x*_4_	0.2	0.2	0.2	1	0.7	0.4
*x*_5_	0.2	0.3	0.2	0.7	1	0.5
*y*	0.2	0.25	0.4	0.4	0.5	1

The first panel of Fig 4 shows the area of overlap of *x*_5_ with the other four independent variables by regressing *x*_5_ on the other independent variables: $R_{i∙1,2\cdots (i)\cdots k}^{2}$. The overlap is .477, which means that the proportion of the variance of *x*_5_ that is linearly independent of the other independent variable is .523. This leads again to two common measures of multicollearity: tolerance: $1-R_{i∙1,2\cdots (i)\cdots k}^{2}$ and the variance inflation factor $1/(1-R_{i∙1,2\cdots (i)\cdots k}^{2})$ or in terms of the diagrams (*area a* = .523) and (1/*area a* = 1.91), respectively. The smaller the independent variance, *area a*, is; the smaller the tolerance and the greater the variance inflation factor.

The left-hand side of the second panel shows that the proportion of variance in *y* associated with the independent variables is .365 ($R_{y∙1,2\cdots k}^{2}$), the cross-hatched area: *area b*. In this case the proportion of the variance that is not accounted for in *y* $\left(1-R_{y∙1,2\cdots k}^{2}\right)$ is .635 (= 1 − .365): *area c*. The diagram on the right-hand side of this panel shows two components of interest. When we regress the dependent variable on all of the independent variables except for *x*_5_, we find that the proportion of variance in *y* accounted for by the four other independent variables is .271: $R_{y∙1,2\cdots (i)\cdots k}^{2}$. This means that the proportion of variance in *y* accounted for by *x*_5_ uniquely is .094 (= .365 − .271). This is labelled *area d* and is the increment in the proportion of variance in *y* accounted for by adding *x*_5_ to a model that contains the other independent variables: $R_{y∙1,2\cdots k}^{2}-R_{y∙1,2\cdots (i)\cdots k}^{2}$. Below, I use these four labelled areas from the modified Venn diagrams to show how these areas relate to the most commonly presented results from standardized and unstandardized regression analyses. The squared standardized regression coefficient for the *x*_5_ is:

$$\beta_{yi∙1,2\cdots (i)\cdots k}^{2}=\frac{R_{y∙1,2\cdots k}^{2}-R_{y∙1,2\cdots (i)\cdots k}^{2}}{1-R_{i∙1,2\cdots (i)\cdots k}^{2}}=\frac{area\;d}{area\;a}=\frac{.094}{.523}=.179.$$ (6)

The absolute value of the standardized regression coefficient (the square root of $\beta_{yi∙1,2\cdots (i)\cdots k}^{2}$) is .423. We can use the *F*-test to determine the statistical significance of this result (the null hypothesis is that the coefficient is zero in the population), and for this example I will assume the correlation matrix is based on 106 cases:

$$F(1,n-k-1)=\frac{R_{y∙1,2\cdots k}^{2}-R_{y∙1,2\cdots (i)\cdots k}^{2}}{\left(1-R_{y∙1,2\cdots k}^{2})/(n-k-1\right)}=\frac{area\;d}{area\;c/(n-k-1)}=\frac{.094}{.635/100}=14.80.$$ (7)

The standard error is:

$$SE(\beta_{y2∙1})=\sqrt{\frac{\left(1-R_{y∙1,2\cdots k}^{2})/(n-k-1\right)}{1-R_{i∙1,2\cdots (i)\cdots k}^{2}}}=\sqrt{\frac{area\;c/(n-k-1)}{area\;a}}=\sqrt{\frac{.635/100}{.523}}=\sqrt{.012}=.1102.$$ (8)

The confidence interval is then constructed by looking up a critical value in a *t*-table with the appropriate degrees of freedom and alpha level and calculating:

$${\beta_{yi∙1,2\cdots (i)\cdots k}\pm t}_{crit}SE(\beta_{y2∙1}).$$ (9)

Both the *t*-test and *F*-test give the same result in terms of statistical significance when there is one degree of freedom associated with the numerator, in which case $t=\sqrt{F}$. To show this is the case, we divide the standardized regression coefficient by its standard error to produce the calculated value of *t* and then compare this to the *F*-test, we use to test for the statistical significance of the regression coefficient:

$$t=\frac{\sqrt{\frac{area\;d}{area\;a}}}{\sqrt{\frac{area\;c/(n-k-1)}{area\;a}}}=\frac{\sqrt{area\;d}}{\sqrt{area\;c/(n-k-1)}}=\sqrt{F}=\sqrt{\frac{.094}{.635/100}}=3.847.$$ (10)

Typically the output from a multiple regression analysis includes the regression coefficients, their standard errors, their *t*-values, and confidence intervals. All of these are presented above and their components presented diagrammatically (except for critical values of *t* and *F*).

We can transform the results to unstandardized values by multiplying the standardized regression coefficients by *sd*_*y*_/*sd*_*i*_ where *sd*_*y*_ is the standard deviation of the dependent variable and *sd*_*i*_ is the standard deviation of the *i*th independent variable:

$$b_{yi∙1,2\cdots (i)\cdots k}=\frac{{sd}_{y}}{{sd}_{i}}\sqrt{\frac{R_{y∙1,2\cdots k}^{2}-R_{y∙1,2\cdots (i)\cdots k}^{2}}{1-R_{i∙1,2\cdots (i)\cdots k}^{2}}=}\frac{{sd}_{y}}{{sd}_{i}}\sqrt{\frac{area\;d}{area\;a}}.$$ (11)

The standard error for the unstandardized regression coefficients is derived similarly:

$${SE}_{b_{i}}=\frac{{sd}_{y}}{{sd}_{i}}\sqrt{\frac{\left(1-R_{y∙1,2\cdots k}^{2})/(n-k-1\right)}{1-R_{i∙1,2\cdots (i)\cdots k}^{2}}}=\frac{{sd}_{y}}{{sd}_{i}}\sqrt{\frac{area\;c/(n-k-1)}{\;area\;a}}.$$ (12)

The *F*-test, *t*-test, and various *R*^2^ values are the same whether we use standardized or unstandardized regression analysis, while the regression coefficients and their standard errors differ depending on whether standardized or unstandardized regression is used.

One other set of statistics that are frequently accompany the output from regression analysis programs is the analysis of variance of regression table (Table 2). The figure corresponding to this table is Fig 4.

**Table 2:** Analysis of variance of regression.

Source	SS	df	MS	*F*(*k*,*n* − *k* − 1)
Model	Σ*y*^2^ × (*area b*) ${\Sigma y^{2}\times (R}_{y∙1,2\cdots k}^{2})$	*k*	(Σ*y*^2^ × *area b*)/*k* ${(\Sigma y^{2}\times R}_{y∙1,2\cdots k}^{2})/k$	$\frac{(area\;b)/k}{\left(area\;c)/(n-k-1\right)}$
Error	Σ*y*^2^ × (area *c*) ${\Sigma y^{2}\times (1-R}_{y∙1,2\cdots k}^{2})$	*n* − *k* − 1	(Σ*y*^2^ × area *c*)/(*n* − *k* − 1) $\frac{\Sigma y^{2}\times (1-R_{y∙1,2\cdots k}^{2})}{n-k-1}$	
Total	Σ*y*^2^ × (1.00) = Σ*y*^2^	*n* − 1	Σ*y*^2^/(*n* − 1)	

Multiplying the areas by the Σ*y*^2^ provides the unstandardized sums of squares accounted for (model sums of squares) and not accounted for (error sums of squares) in *y* and the total sums of squares. The mean squares are the sums of squares divided by the corresponding degrees of freedom, and *F* is the mean square associated with the model divided by the mean square for error. The Σ*y*^2^ cancel each other out in the computation of *F* in the final column. This *F*-test has a null hypothesis that the independent variables in the model account for none of the variance in *y* in the population and has *k* degrees of freedom associated with the numerator. The *F*-test used to test for the significance of a single partial regression coefficient (Eq 7) had only one degree of freedom associated with the numerator.

## Discussion

The use of Venn diagrams has been suggested in the literature because of they allow students and researchers to “see” diagrammatically many or the key components in multiple regression [1,4,5,10]. These diagrams are not seen as a replacement for the algebra (or calculus or matrix algebra) associated with regression analysis, but as an additional tool to help students and researchers gain a better intuitive understanding of these methods. In the multiple regression context; the traditional Venn diagram approach is helpful in some cases, but misleading in others.

Problems arise for the traditional Venn diagram approach used in statistics when there are two or more independent variables. In this situation the traditional Venn diagram approach fails to adequately represent many components as areas of overlap for the variables in the Venn diagrams: and it is these overlaps that are essential to understanding visually the components of multiple regression. Because of these types of problems some suggest doing away with the traditional Venn diagram representations. Hunt [11] has sections in his article on the design of ballentines entitled “What Ails Ballentines Representing Partial and Multiple Correlations and another entitled, “Depicting Suppressor Variables: A Fatal Ailment.” (The “ballentine” is an alternative expression for the traditional Venn diagram approach used in statistics.) Fox [12] notes that the overlaps in Venn diagrams would have to be negative to adequately represent some situations.

As an alternative, I propose a modified Venn diagram approach that considers only the overlap between two Venn circles at any one time. It represents the overlap of two or more independent variables with the dependent variable with only a single circle for those independent variables and the overlap of multiple independent variables with another independent variable with only a single circle used to represent the multiple independent variables. The circles are of equal size to represent the fact that a combination of variables can account for a proportion of variance in the target variable that ranges from 0.00 to 1.00.

I am not the first to use a single circle to represent the combined relationships of several variables with another variable, but the insistence on using only two-circle Venn diagrams, the reasoning behind using equal size circles, and the concept of a target variable are new (certainly new in combination with one another). The modified Venn diagram approach allows a consistent diagrammatic representation of shared variance for an independent variable with other independent variables and for the dependent variable with independent variables.

This approach allows students to have a consistent diagrammatic representation of regression coefficients, their standard errors, and *F*-tests and *t*-tests that determine their statistical significance. There is more that can be represented, but I leave other extensions to the interested reader. Many of these extensions will be straightforward using the approach taken in this paper.
